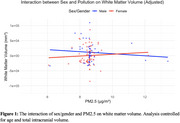# The role of Air‐Borne Pollutant exposure on Brain and cognitive aging

**DOI:** 10.1002/alz70856_102446

**Published:** 2025-12-24

**Authors:** Sophia Licata, Valentina E. Diaz, Carina Lo, Anna M. VandeBunte, Rowan Saloner, Shubir Dutt, Coty Chen, Linh Pham, Molly Olzinski, Emily W. Paolillo, Lana S. Callies, Savannah R. Hallgarth, Clare Andriola, Joel H Kramer, Kaitlin B Casaletto, Claire J. Cadwallader

**Affiliations:** ^1^ University of California San Francisco, San Francisco, CA, USA; ^2^ Memory and Aging Center, UCSF Weill Institute for Neurosciences, University of California, San Francisco, San Francisco, CA, USA; ^3^ Memory and Aging Center, Weill Institute for Neurosciences, University of California San Francisco, San Francisco, CA, USA

## Abstract

**Background:**

Air‐borne pollutants are increasingly prevalent and may be an important risk factor for cognitive aging and dementia. Preliminary evidence suggests air pollution is adversely associated with brain health outcomes in older adults, including cognitive function and total grey and white matter volumes. Understanding person‐specific factors that increase vulnerability to pollutants is important for dementia prevention strategies. The present study examined the interaction of air‐borne pollutant exposure with age and sex on cognitive and brain imaging outcomes in cognitively unimpaired older adults.

**Methods:**

258 older adults (mean age = 66 ± 9.6, 56% female) enrolled in the UCSF Brain Aging Network for Cognitive Health study had zip code data for air pollution exposure estimates and completed a brain MRI and neuropsychological testing. Primary outcomes included cognitive composite scores (memory and executive function), total grey matter (GM) and white matter (WM) volume. Levels of PM2.5 concentration and toxin release (toxicity weighted concentrations of chemical releases) in the San Francisco Bay Area were derived via residential ZIP codes from the Office of Environmental Health Hazard Assessment. Partial correlations examined the association of pollutants with cognitive and brain outcomes controlling for sex, age, education, and total intracranial volume. Separate linear regression models then evaluated the interaction of pollutants with age and sex on outcomes.

**Results:**

Direct associations between air pollutants with brain health outcomes were small to minimal (partial r range = ‐0.15 to ‐0.02, *p*‐values>0.102). Interaction models revealed that relationships between pollutants and outcomes of interest did not differ by age. In contrast, there was a significant interaction between PM2.5 and sex, such that the negative relationship between PM2.5 (particulate matter) and WM volume was stronger in males compared to females (b=15970.00, *p* = 0.048). Interaction models examining PM2.5 and sex in relation to cognitive outcomes or total GM volume did not reach statistical significance.

**Conclusion:**

Living in areas with greater concentrations of PM2.5 was associated with lower WM volume in older males compared to females. These findings suggest that the male brain may be more vulnerable to air‐borne pollutive agents. Further research is needed to understand the mechanism of this sex‐specific sensitivity.